# 2024 Mpox outbreak: A rapidly evolving public health emergency of international concern: Introduction of an Updated Mpox Identify-Isolate-Inform (3I) Tool

**DOI:** 10.1016/j.onehlt.2024.100927

**Published:** 2024-11-07

**Authors:** Aileen M. Marty, Christian K. Beÿ, Kristi L. Koenig

**Affiliations:** a Herbert Wertheim College of Medicine, Department of Translational Medicine, and College of Arts, Sciences, and Education and Florida International University, Miami, Florida, USA; b School of Medicine, University of California San Diego, La Jolla, California, USA; c County of San Diego, Emergency Medical Services Office, Public Safety Group – San Diego County Fire, San Diego, California, USA; d Departments of Emergency Medicine and Public Health, University of California Irvine, Orange, California, USA

**Keywords:** Mpox, Monkeypox, PHEIC, Clades Ia, IIa, Ib, IIb, Outbreak, Identify-isolate-inform, 3I tool, Smallpox, Zoonotic virus, Climate change, Orthopoxvirus, Transmission, Clinical, Treatment

## Abstract

The declaration of a second Public Health Emergency of International Concern (PHEIC) for mpox in August 2024 underscores the urgent need for a comprehensive understanding of the evolving epidemiology [1] clinical manifestations, and zoonotic potential of this emerging threat [2]. This work delves into the intricate interplay between human and animal mpox infections, with a specific focus on the unique characteristics of various viral clades and their implications for individual and public health.

There is a critical need to elucidate the factors driving multiple spillover events and the subsequent emergence of new clades better adapted to human-to-human transmission. We hypothesize that anthropogenic changes, including deforestation, urbanization, and climate change are facilitating increased human-to-animal contact, leading to more frequent zoonotic transmissions and viral adaptations. Our conceptual framework integrates One Health principles, evolutionary virology, and epidemiological modeling to investigate the demographic, clinical, and treatment differences among mpox clades in both humans and animals. We employ a mixed-methods approach, combining genomic analysis, clinical data review, and ecological surveys to construct a comprehensive picture of mpox's changing dynamics. The research questions explore the differences in epidemiological and clinical profiles among mpox clades and the factors that likely contribute to successful cross-species transmission and human adaptation.

This manuscript introduces an updated Identify, Isolate, Inform (3I) Tool meticulously redesigned to significantly improve the early detection, containment, and reporting of mpox cases across diverse settings. By integrating clinical, virological, and ecological data, this work aims to lay the groundwork for enhanced risk assessment, targeted interventions, and global preparedness strategies in the face of this evolving zoonotic threat.

## Highlights

• The paper reviews the One Health aspects of mpox/monkeypox.
• The epidemiology of this virus has continued to evolve since 2022, prompting a second mpox Public Health Declaration of International Concern (PHEIC).
• This paper describes the demographic and epidemiologic risk factors alongside the clinical manifestations of the four known mpox clades (Ia, IIa, Ib, IIb).
• Transmission, correlation with climatic and anthropomorphic factors, outbreak prevention and management strategies, treatment, and testing are presented.
• The work updates the Mpox Identify-Isolate-Inform (3I) Tool for application to the 2024 public health emergency.

## 1 Introduction

The World Health Organization (WHO) declared its eighth Public Health Emergency of International Concern (PHEIC) on 14 August 2024 [3]. This is the second time that disease by mpox virus, an orthopoxvirus that causes mild to severe constitutional symptoms and produces a rash with lesions that resemble those of smallpox [4], has become a PHEIC, the first being from 2022 to 2023. Clade IIb, responsible for the 2022 outbreak, continues to spread in Europe, the Americas, and Asia. In 2024, a new clade Ib, was detected and there have been sustained and increased transmission of zoonotic clades of MPXV. These developments, coupled with inadequate quantities and distribution of medical countermeasures, required a new declaration.

This alarming sudden rise and expanded distribution of mpox in 2024, especially of the more pathogenic clade I, includes cases documented not only in the Democratic Republic of the Congo (DRC), where it is endemic, but also in Kenya, Rwanda, Burundi, Uganda, South Sudan, Côte d'Ivoire, Sweden [5], and Thailand [6]. There is a complex interplay of genetic, ecological, epidemiological, physiological, and immunological factors, all exacerbated by anthropogenic environmental changes and shifting behaviors. This is a One Health issue because ecological changes, such as deforestation, urbanization, and increased ecotourism have heightened human-wildlife interactions, leading to more spillover events, which have led to altered viral transmission.

This paper describes the impact of anthropomorphically related global changes on the shifting epidemiology of mpox as it pertains to humans and animals. Transformed ecosystems, driven by rising carbon dioxide (CO2) levels and global warming, have shifted the range of reservoir hosts, including *Funisciurus* spp. and *Heliosciurus* spp. Improved transportation infrastructure is facilitating the spread. The detection of clade Ib in travelers to Sweden and Thailand [7] highlights how global travel and trade facilitate the virus's international distribution. Lack of herd immunity combined with limited availability of vaccines like MVA-BN and ACAM2000, particularly in Africa, exacerbated by vaccine hesitancy, political and economic factors, and logistical challenges in distribution contribute to the rapid spread of mpox. The spillovers have allowed MPXV to develop genetic adaptations that enhance its replication and transmission in humans.

Clinicians must distinguish cases of mpox not only from other infections but also, ideally, differentiate MPXV clade Ia, Ib, IIa, and IIb. The clades have different demographic distributions, rates of person-to-person transmission, incubation periods, disease severity, and response to therapy. While general mpox polymerase chain reaction (PCR) is widely available, clade-specific nucleic acid amplification tests (NAATs) or sequencing are necessary for precise identification and definitive differentiation.

This manuscript updates the Mpox Identify-Isolate-Inform (3I) Tool. After an initial assessment of risk factors and signs/symptoms, patients are identified as potentially exposed or infected. Management of exposed patients includes separating them from vulnerable individuals, supportive, immunologic, and antiviral treatment, and post-exposure prophylaxis via vaccination. Outbreak management involves ring vaccination, dissemination of information, destigmatization, and rumor control. Healthcare workers must report suspected and confirmed cases in humans or animals to public health authorities or the World Organization for Animal Health (WOAH). The updated 3I Tool will aid emergency, primary care, and prehospital clinicians to effectively manage individuals with suspected or confirmed mpox.

## 2 Epidemiology and background

Initial recognition of MPXV as separate from smallpox was accomplished using non-molecular methods that could not distinguish between clades. Early research on mpox from the 1970s to the 1990s was limited in part because mpox was considered a sporadic, zoonotic, uncommon human disease that required high biosafety measures. Additionally, limited funding and available sequencing methods were much less advanced and much more expensive, particularly for analyzing the genome of a large and complex DNA virus, such as MPXV. Thus, while some preliminary genetic information had been gleaned using comparative analysis of restriction endonuclease site maps plus short DNA sequences, those studies had only provided insights into 7 % of the MPXV genome.

The increasing awareness of orthopox viruses [8], including mpox [9] as a potential biothreats, helped create funds [10] that allowed researchers in 2001 to sequence MPXV comprehensively. Shchelkunov and colleagues used a sample collected during the 1996 mpox outbreak in the DRC (then called Zaire) and established the genome of the Central African Clade; their work also demonstrated that while MPXV-ZAI was at least 96.3 % identical to variola (smallpox) virus (VAR), there were considerable differences in the regions encoding virulence and host-range factors near the ends of the genome [11].

Recognition that a different clade was responsible for mpox outbreaks in West Africa followed the importation of MPXV from Ghana to the United States (US) in 2003. The small zoonotic US outbreak allowed researchers from the US Centers for Disease Control and Prevention (CDC) to identify and deposit the clade II MPXV sequence in GenBank in 2004 and publish it in 2005 [12]. Thus, two distinct variants of MPXV based on genetic and geographic differences were recognized: the “Central African Clade” (clade I) and the “West African Clade” (clade II).

**Fig. 1:**
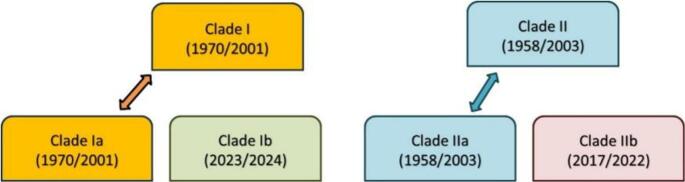
A visual representation of mpox clades showing year isolated/year sequenced.

MPXV is currently classified into two distinct clades (I and II) *and* four distinct subclades: Ia, Ib, IIa, and IIb (Fig. 1), each with unique characteristics and epidemiological patterns [13].

### 2.1 Clade Ia

MPXV was first comprehensively sequenced in 2001. The isolate MPXV (NC_003310.1) came from the DRC and is the same clade as that from the first human case (1970, DRC) [14]. It is endemic in Central Africa and exhibits high genetic diversity [15]. It predominantly affects children under 15 years of age and has a relatively high mortality rate of approximately 5–10 % [16].

### 2.2 Clade IIa

The subclade currently designated clade IIa was identified during the 2003 US zoonotic outbreak linked to prairie dogs (the prairie dogs had contracted the virus from infected Gambian giant rats imported from Ghana) [17]. Curiously, the first MPXV ever identified, the Copenhagen strain (MPXV-COP-58), found in non-human primates (NHP) in 1958, was clade IIa [18]. Clade IIa shows less genetic diversity compared to Ia and is primarily associated with zoonotic transmission. Clade IIa predominantly affects adults, with the primary rash typically appearing at the site of animal contact. No deaths have been reported from IIa.

### 2.3 Clade IIb

The re-emergence of MPXV in Nigeria in 2017 [19] led to the sequencing and deposition of the subclade IIb in GenBank 2017 [20]. However, it was not recognized as a new subclade until the 2022 global outbreak. Thus, not only genetic but also epidemiological and clinical data led researchers to recognize that the West African clade had evolved into two subclades, IIa and IIb [21]. With IIb, person-to-person rather than zoonotic spread became the dominant form of transmission, mainly through sexual contact [22], and primarily affecting adults, especially men who have sex with men. Human-to-human transmission with rapidly rising international cases led the WHO to declare the first mpox PHEIC from July 2022 to May 2023 [23].

### 2.4 Clade Ib

In April of 2023, a Belgian man visiting the DRC tested positive for clade I in Kenge, Kwango Province, and his sexual contacts also tested positive for clade I. Before April 2023, there were no formally documented cases of sexual transmission of clade I MPXV. In September 2023, Kamituga, DRC, reported its first-ever mpox cases, mostly among young adults and sex workers. After several more clusters of sexually transmitted clade I, samples from a set of sex workers were sequenced [24]. In January 2024, results detected a new subclade, Ib. [25] Characterized by sustained and increased human-to-human transmission, Ib is primarily observed in young men and women, but also affects children. It commonly presents with genital lesions and is mainly transmitted through sexual contact, including heterosexual sex.

A comprehensive synthesis mpox epidemiologic features is presented in Table 1A.

**Table 1A:** Epidemiological characteristics of MPXV clades [26].

Epidemiological characteristics	Clade Ia	Clade Ib	Clade IIa	Clade IIb
Initial Identification	• Caused the first known human case in 1970 in the DRC • First sequenced in 2001	• DRC described sexually transmitted cases of clade I in 2023 • Sequencing from sex workers in January 2024 identified Ib	• Isolated from non-human primates in 1958 • Sequenced from cases of the 2003 US zoonotic outbreak imported from Ghana	• Produced a resurgence of mpox in Nigeria in 2017 and sequenced same year • Not recognized as a new clade until 2022
Global Distribution	• Endemic in Central Africa	• Primarily in Central Africa, affecting multiple African nations • Cases have spread by travelers to Thailand and Sweden	• Sporadic cases are mainly linked to animal contact or travel	• Responsible for the ongoing 2022 global outbreak, widespread internationally • New sub-lineages (e.g., C.1.1) continue to emerge
Transmission	• Significant zoonotic transmission • Limited nosocomial / familial spread	• Increased human-to-human transmission, mainly through sexual contact, including female sex workers • Nosocomial / familial spread	• Primarily zoonotic transmission • Limited nosocomial / familial spread	• High human-to-human transmission, mainly through sexual contact of men having sex with men • Limited nosocomial / familial spread

## 3 Virology and molecular biology

All MPXV clades share fundamental virological characteristics (Table 1B). They are large, enveloped rectangular virions with a complex internal structure containing a nucleocapsid core and lateral bodies. The core has a genome of linear double-stranded DNA of approximately 196 to 197 kb (range 195 to 200 kb) that encodes about 190 genes. The ends of the genome have inverted terminal repetitions (ITR), which are identical but reversed sequences. Core proteins include enzymes and other proteins needed for early gene expression and DNA replication. Genes for transcription, replication, and virion assembly (e.g., polymerase, capping enzymes, polyadenylation factors) are in the central area. These genes are necessary since MPXV replicates in the cytoplasm.

**Table 1B:** Virological characteristics of MPV.

Characteristic virological	Clade Ia	Clade Ib	Clade IIa	Clade IIb
Molecular Characteristics	• Exhibits a high degree of nucleotide diversity • APOBEC3 mutations present but at a lower frequency (8–13 % of mutations)	• Higher nucleotide diversity compared to Ia • Mutations identified in APOBEC3 indicative of increased human-to-human transmission	• APOBEC3 mutations present but at a lower frequency (8–13 % of mutations) • Similar to Ia	• Significant APOBEC3-driven mutations continue to accumulate • 50–60 % of mutations are APOBEC3-style
APOBEC3 Mutation Patterns	• Low frequency of TC → TT and GA → AA mutations • Consistent with standard models of nucleotide evolution	• Increased frequency of APOBEC3-style mutations compared to Ia, but lower than IIb	• Low frequency of TC → TT and GA → AA mutations • Similar to Ia	• High frequency of TC → TT and GA → AA mutations, particularly in the IIb B.1 lineage
Genomic Evolution	• Slower accumulation of mutations compared to IIb	• Intermediate rate of mutation accumulation	• Slower accumulation of mutations, similar to Ia	• Rapid accumulation of mutations, particularly APOBEC3-driven changes • New sub-lineages (e.g., IIb C.1.1) emerging with unique mutations

**Fig. 2:**
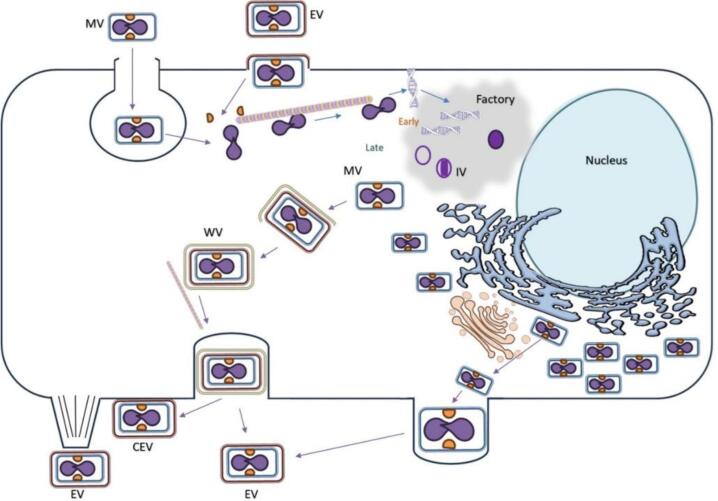
The lifecycle of MPXV in a host cell. CEV: cell-associated enveloped virus; IV: immature virion; MV: mature virion. (Illustration by Dr. Aileen Marty).

The life cycle involves two infectious forms: the extracellular virion (EV) and the mature virion (MV). EVs are MVs with an additional membrane and slightly different surface proteins in the inner membrane [27]. The MV binds cells and then enters via micropinocytosis (Fig. 2). An EV must drop its outer envelope and enter via fusion. The viral core uses microtubules to reach perinuclear regions of the cytosol, where the virus replicates in “viral factories” surrounded by endoplasmic reticulum membranes. The number of factories correlates with the amount of infection per cell, albeit factories can coalesce.

Immunomodulating proteins in lateral bodies reduce innate host defenses. Early genes are expressed, which mediate the uncoating of the core. Early genes synthesize early viral mRNA, which code for enzymes and other factors needed for the synthesis of viral DNA and transcription of intermediate genes. The intermediate genes encode enzymes and factors for late gene expression. The products of the late genes include transcription factors, viral RNA polymerase, and other enzymes and structural proteins that produce the progeny virions. Crescents enclose viral core proteins and genomic DNA, forming immature virions (IV). The IVs mature into MVs. A small percentage of MVs are transported to the Golgi/endosomal compartment, where they are wrapped with a double membrane to produce wrapped virions (WV). The WVs use microtubules to transport to the cell surface and can exit as EVs. The virus can exit either intact cells when WVs exit as EVs or as MVs when cells lyse.

### 3.1 Drivers of MPOXV evolution

APOBEC3 (Apolipoprotein B mRNA-editing catalytic polypeptide-like 3) proteins are crucial for defending against various viral infections. Humans and other primates have 7 isoforms of APOBEC3 (A, B, C, D, F, G, and H) with slightly different functions, but all are host enzymes critical to the innate immune system. While some APOBEC3 enzymes are constitutively expressed in many cells, they can also be induced by the binding of interferons to their receptors and the activation of signaling pathways that enhance transcription factors, awakening the APOBEC3 genes that synthesize APOBEC3 enzymes.

The goal of these enzymes is to function as restriction factors against retrotransposons, retroviruses, and some DNA viruses by inducing hypermutation in viral genomes. Ironically, research reveals that APOBEC3-driven mutations seem to be promoting the evolution of MPXV [28], albeit at different rates in the different subclades. In particular, APOBEC3-F is specifically potent at extensively deaminating cytidine residues in MPXV genomes and most of the observed MPXV mutations of clade IIb are related to the actions of APOBEC3-F. [29] Moreover, G → A hypermutated MPXV genomes were recovered experimentally from APOBEC3-F transfection followed by MPXV infection.

A review of the MPXV isolate sequences in GenBank reveals that Ib has many scattered mutations, mainly single-nucleated polymorphisms (SNPs), but also a few deletions and insertions, that distinguish it from Ia. These mutations involve genes that alter the transmission and pathogenicity of MPXV and include mutations to the viral DNA polymerase gene, RNA polymerase subunit genes, envelope protein genes, and immune evasion genes.

Key viral mutations, such as those in the F13L gene (responsible for viral egress), A35R (immune modulation), and B8R (suppression of T cell activation) [30], have significantly enhanced the virus's ability to spread among humans. Notably, some mutations in F13L have also reduced the effectiveness of tecovirimat [31]. A substantial ∼1 kb deletion (Δ19,128–20,270 coordinates) identified in the Ib genomes in GenBank for MPXV appears to have altered viral protein expression and immune-evasion strategies [32]. Mutations induced by the mammalian enzyme APOBEC3, such as TC → TT and GA → AA, are more prevalent in “b” clades than the “a” clades (IIb compared to IIa and in Ib compared to Ia.)

Clades Ia and IIa exhibit a lower frequency (8–13 %) of these mutations, while IIb shows a significantly higher frequency (50–60 %), and Ib also has a higher frequency [33]. This increased rate of APOBEC3-style mutations in Ib and IIb are associated with their rapid evolution and increased human-to-human transmission [34]. Thus, this defense mechanism is ironically fueling the rise of mpox.

### 3.2 Clinical manifestations in humans

#### 3.2.1 Transmission

MPXV must enter through a mucosal surface or broken skin. Viruses cannot replicate in dead cells; thus, intact, healthy, keratinized skin is not permissive to viral entry. Mucosal surfaces include conjunctival, genital, respiratory (nasal), and enteric (oral/anal) surfaces. Generally, transmission requires prolonged close contact that permits viral entry through a mucosal surface. Nevertheless, airborne transmission is feasible and documented for other orthopoxviruses. The data indicate that transmission of the “a” clades is generally by spillover from animals to humans; the “b” clades are more readily transmitted person-to-person, mainly by sexual contact. Additional human-to-human transmission is via close contact with cough, bodily fluids, or active skin lesions, and viral particles from these sources can contaminate clothing, bed sheets, and other household items, which can serve as sources of household or nosocomial infections. Mothers can transmit infection congenitally. Humans can transmit to animals; animals can transmit to humans or other animals [35].

#### 3.2.2 Incubation period

The incubation periods vary slightly among clades. Clade Ia has a mean incubation period of 12 days (range 7 to 31 days), Ib 8.1 days (range 3 to 17 days), IIa 12 days (range 1 to 31 days), and IIb 9.5 days (range 3 to 34 days). Some people are contagious during the incubation period, i.e., prior to symptom onset.

For Ia and IIa MPXV infections, the contagious period is generally from the onset of prodromal symptoms until all scabs have fallen off and a fresh layer of skin has formed [36]; this typically lasts 2–4 weeks. For IIb, the contagious period often precedes symptom onset by 1 to 2 days and continues until all scabs have fallen off and a fresh layer of skin has formed [37]. Importantly, patients with IIb can be contagious and never develop symptoms [38]. The contagious period of Ib is unknown.

#### 3.2.3 Signs and symptoms

Infected persons can be asymptomatic, but most develop clinical manifestations, with signs and symptoms varying somewhat among the clades (Table 1C). Patients with Ia classically manifest prodromal constitutional symptoms (fever, chills, headache, myalgia, exhaustion, and lymphadenopathy) before the enanthem and exanthem (skin rash). Patients with the “b” clades may not show any prodromal symptoms, or the constitutional symptoms may develop simultaneously to an enanthem or exanthem or after the rash. These systemic symptoms in IIb often lack fever or lymphadenopathy. Many IIb patients have a mild, self-limiting illness that does not require antiviral treatment. (See Table 2.)

**Table 1C:** Clinical presentation of MPXV.

Characteristics clinical presentation	Clade Ia	Clade Ib	Clade IIa	Clade IIb
Incubation Period	• Mean incubation of 12 days (range 7 to 31 days)	• Mean incubation of 8.1 days (range 3 to 17 days)	• Mean incubation of 12 days (range 1 to 31 days)	• Mean incubation of 9.5 days (range 3 to 34 days)
Contagious period	• With the onset of prodrome until the last scabs fall off and a fresh layer of skin has formed	• The onset is not yet known • Likely contagious until the last scabs fall off and a fresh layer of skin has formed	• With the onset of prodrome until the last scabs fall off and a fresh layer of skin has formed	• 1–2 days before symptom onset (constitutional symptoms, enanthem, or exanthem) • Continues until all lesions have crusted, scabs have fallen off, and a fresh layer of skin has formed
Clinical Characteristics	• Predominantly affects children <15 years • Prodrome: fever and other constitutional symptoms present before the enanthem (mucosal rash), followed by exanthem (Skin rash) • Rash is primarily on the face, with centrifugal distribution [face → extremities (including palms/soles) → trunk], and shows regional monomorphism • Lymphadenopathy is typical	• Predominantly affects young adults • High prevalence of genital lesions	• Predominantly affects adults • Primary rash at the site of animal contact lesions with regional monomorphism	• Predominantly affects adults, especially men who have sex with men • Primary rash in the anogenital region • Constitutional symptoms may not exist or precede, follow, or be concurrent with a rash • Lesions may show regional pleomorphism • Rash can be localized to genital, perineal, perianal, or oral areas • Anorectal pain or tenesmus can be severe.
Mortality and Morbidity	• High mortality rate (5–10 %); Complications include encephalitis, ocular disease, secondary infections of skin or lungs (pneumonia)	• The mortality rate is unknown, but it is suspected to be lower than that of Ia	• No reported deaths	• Lower mortality rate (0.04 % to 0.19 %) than Ia, but higher than IIa
Diagnostic Tools	• Recent advancements in diagnostic tools with high sensitivity and specificity for Clade I and II • Limited clinical performance data is available	• Similar diagnostic advancements are applicable • Limited clinical performance data is available	• Similar diagnostic advancements are applicable • No clinical performance results were reported	• Lab-based PCR tests show 95–100 % sensitivity and 100 % specificity • Point-of-care molecular tests show 89–100 % sensitivity and 100 % specificity

**Table 2:** Summary of MPXV in animals. NAAT stands for Nucleic Acid Amplification Test, which includes PCR testing. The symptomatology for many species is listed as “Unknown” when evidence of whether they are typically symptomatic or asymptomatic when infected with MPXV is lacking.

Animal	Identification Method	Symptomatology	Reference
African brush-tailed porcupine (*Atherurus africanus*)	Serological	Unknown	Parker, 2013 [47]
African hedgehog (*Atelerix* sp.)	NAAT	Unknown	Parker, 2013
Apes • Chimpanzee *(Pan troglodytes)* • Gorilla (*Gorilla gorilla*) • Orangutan (*Pongo pygmaeus*)	Serological, NAAT	Symptomatic (skin lesions, fever, severe respiratory symptoms, High mortality)	Patrono, 2020 [48] Parker, 2013
Chinchilla (*Chinchilla* sp.)	Unknown	Unknown	CDC, 2023 [49]
Dog (*Canis lupus familiaris*)	NAAT	Symptomatic (skin lesions, mucocutaneous lesions)	Seang, 2022 [50]
Dormouse (*Graphiurus* sp.)	NAAT	Unknown	Parker 2013.
Gambian pouched rat (*Cricetomys gambianus*)	Serological, NAAT	Likely asymptomatic, shed high viral loads from multiple routes	Hutson, 2015 [51] Falendysz, 2023 [52]
Giant anteater (*Myrmecophaga tridactyla*)	Unknown	Symptomatic (fever, skin lesions)	CDC, 2023 Parker 2013.
Jerboa / Dipodoid rodents (*Jaculus* sp.)	NAAT	Unknown	Parker, 2013.
Marmot/Woodchuck (*Marmota* sp.)	Unknown	Unknown	CDC, 2023
Monkeys • Cynomolgus macaque (*Macaca fascicularis)*, • Rhesus macaque (*Macaca mulatta*), • Sooty mangabey (*Cercocebus atys*)	Serological, NAAT	Symptomatic (skin lesions, fever, lymphadenopathy)	Gispen, 1976 [53].
Opossum, • Gray short-tailed Opossum • (*Monodelphis domestica*) • Southern Opossum, (*Didelphis marsupialis*)	Virus isolation, NAAT	Unknown	Parker, 2013
Prairie dog (*Cynomys ludovicianus*)	Epidemiological, NAAT	Symptomatic (skin lesions, respiratory symptoms, fever, lethargy)	Xiao, 2005 [54] Hutson, 2009
Shrew (various genera and species)	Unknown	Unknown	CDC, 2023
Squirrel, Sun (*Heliosciurus rufobrachium*)	Serological, NAAT	Symptomatic (skin lesions)	Parker, 2013
Squirrel, Thomas's Rope (*Funisciurus anerythrus*)	Serological, NAAT	Symptomatic (skin lesions, fever, lymphadenopathy)	Falendysz, 2023
Squirrel, Tree (*Heliosciurus* sp.)	Serological, NAAT	Symptomatic (skin lesions)	Parker, 2013
Woodchuck (*Marmota monax*)	NAAT	Unknown	Parker, 2013

Patients with mpox can have painful lesions requiring analgesia. When lesions involve the oral cavity or pharynx, they can lead to problems with dehydration and nutrition. Painful lesions affecting the anogenital area can cause issues with urination or defecation. Some patients develop short or long-term sequelae, including corneal scarring, obstructive lymphadenitis, sepsis, and secondary infections such as pneumonia or wound infections. Underlying infections with HIV, herpes, and other pathogens can lead to more severe cases of mpox.

#### 3.2.4 Mortality

The mortality rate is highest for clade Ia, ranging up to 11 %. No known IIa cases have resulted in human deaths. The mortality rate for IIb is lower than that of Ia, at approximately 0.04 % to 0.19 %. [39,40] The mortality rate for Ib is not yet known.

A randomized, placebo-controlled study (PALM 007) conducted in the DRC revealed intensive supportive care resulted in a low mortality of 1.7 % [41]. Supportive care included hydration, nutrition, treatment of secondary infections, and monitoring in the hospital for resolution of lesions and management of complications. As of this writing, most MPXV cases in Africa have not been sequenced. Thus, it is uncertain how many of the 582 [42] deaths reported in African nations between January and August of 2024 [43] were the result of infection with clade Ib.

## 4 MPXV in animals

MPXV can cause fever, lymphadenopathy, enanthem, and exanthem in many mammalian species; however, some animals are asymptomatic. MPXV is an amphixenosis, a type of zoonosis that is mutually transmissible between animals and humans [44]. Squirrels are the most likely reservoir host, though the data are inconclusive. Experimental MPXV infection of rope squirrels caused them to shed very high levels of MPXV for 5 to 22 days, and they presented with constitutional symptoms, lymphadenopathy, and rash. They had moderate to severe morbidity and a mortality of 75 % in those infected intranasally [45].

MPXV in prairie dogs resembles clade I MPXV in humans; the animals experience constitutional symptoms (fever, fatigue) during a prodromal period and later develop skin lesions. In experimental studies, prairie dogs developed skin lesions 9 to 12 days after infection with either the Central African clade Ia or the West African clade IIa; however, those infected with IIa recovered, while those infected with Ia had a high mortality rate [46].

As of September 2024, the World Organization for Animal Health (WOAH) has not yet confirmed clade Ib in animals but is asking its members to notify if such an event is detected.

## 5 Diagnostic tools for MPXV

The diagnostic tools for MPXV in humans and other mammals are generally similar, but there are some specific considerations for animal testing. When testing for MPXV, the use of WHO [55] or WOAH safety protocols is indicated. Diagnostic tools for MPXV have advanced significantly for both humans and animals; real-time PCR is the gold standard for MPXV detection.

Some PCR tests use generic primers that target the E9L gene found in orthopoxviruses. General (i.e., clade-nonspecific) MPXV testing is available through commercial laboratories. MPXV-specific primers can target unique MPXV genes to distinguish them from other orthopoxviruses; however, distinguishing clades is challenging since clades I and II share 99 % sequence identity. A difference between clades I and II is in the tumor necrosis factor receptor (*TNFR*) gene, located at the end of the linear DNA in the ITR. The *TNFR* gene has many SNPs and insertion/deletions (indels) that differ between clades I and II [56]. However, those differences do not necessarily distinguish the subclades.

In humans, lab-based PCR tests show 95–100 % sensitivity and 100 % specificity for clade IIb, while point-of-care molecular tests demonstrate 89–100 % sensitivity and 100 % specificity. However, diagnostic data for clade I, particularly Ib, remain limited. Next-generation sequencing rapidly and conclusively distinguishes between clades [57]; however, it is not widely available.

Enzyme-linked immunosorbent assays (ELISA) and neutralization assays are used for both humans and animals [58]. However, there are challenges, such as cross-reactivity with other orthopoxviruses and the need for species-specific secondary antibodies for ELISA tests for some animals. Good immunohistochemical tools are available to diagnose MPXV in tissue samples from both humans and animals.

## 6 Management

### 6.1 Treatment

Supportive therapy includes nutrition, hydration, monitoring for secondary infections (with prompt treatment with appropriate antibiotics or other medications), and close patient monitoring, ideally in a hospital setting. These measures significantly reduce mortality. Supportive treatment can be supplemented with antiviral therapy. Antiviral treatments include tecovirimat (TPOXX), cidofovir, brincidofovir, trifluridine (for eye lesions), NIOCH-14, and IV VIG.

TPOXX inhibits the viral protein 37 (VP37), preventing the formation of wrapped virions (WV) and their egress from cells as EVs [59]. However, resistance to TPOXX has been observed in patients with Mpox in multiple studies. [[60], [61], [62]] Notably, some mpox viruses have mutations in F13L that reduce tecovirimat effectiveness. Moreover, the PALM 007 trial conducted in the DRC showed that TPOXX did not meet the trial's primary endpoint of significantly improving lesion resolution time compared to placebo. However, TPOXX was safe and showed potential benefits for severely infected patients who were treated early. Oral tecovirimat is available in the US for treatment of mpox via the STOMP [63] trial or the CDC's Expanded Access Investigational New Drug (EA-IND) protocol for patients who meet eligibility criteria [64]. NIOCH-14, a tecovirimat analogue under investigation, also inhibits WV formation.

Trifluridine is a thymidine nucleoside analogue antiviral drug that is effective against various DNA viruses, including MPXV [65]. As a 1 % ophthalmic solution, trifluridine can be applied directly to the affected eye.

Cidofovir is a nucleotide analog of deoxycytidine monophosphate (dCMP); it has a phosphonate group (instead of a regular phosphate) attached to the ribose ring. This drug competitively inhibits the incorporation of dCTP into viral DNA by viral DNA polymerase, causing chain termination in viral DNA synthesis [66]. Cidofovir targets viral DNA polymerase broadly and, thus, is mechanistically useful for several DNA viruses. It is FDA-approved for treating cytomegalovirus (CMV) retinitis in patients with AIDS. The US CDC recommends the off-label use of cidofovir for immunocompromised patients with mpox [67] with monitoring of creatinine to detect nephrotoxicity and for drug interactions [68]. Avoid use in patients with serum creatinine with >1.5 mg/dL. Cidofovir is given intravenously along with oral probenecid to improve effectiveness and reduce nephrotoxicity.

Brincidofovir is a lipid conjugate of cidofovir that can be taken orally; it has a lipid side chain (hexadecyloxypropyl) attached to the phosphonate group of cidofovir. This lipid conjugation allows brincidofovir to enter cells more efficiently than cidofovir. Once within cells, it is metabolized to cidofovir, resulting in higher intracellular concentrations. The lipid conjugation prevents brincidofovir from accumulating in the kidneys, significantly reducing the risk of nephrotoxicity compared to cidofovir [69].

Vaccinia Immune Globulin Intravenous (VIGIV) is FDA-licensed for use in smallpox, not for mpox, and there are no published studies of VIGIV use in mpox. However, the CDC has an EA-IND protocol for the use of VIGIV advising clinicians to consider use of VIGIV for persons with severe T-cell deficiency for whom vaccination for orthopox viruses is contraindicated [70].

CDC recommends vaccination as post-exposure prophylaxis (PEP) to those with known or presumed exposure to MPXV. Provide the vaccine as PEP as soon as possible, ideally within four days of exposure, but 4 through 14 days after exposure can provide some protection against mpox. Clinicians can administer MVA-BN to people with risk factors, including those engaging in activities that may expose them to MPXV. [71]

### 6.2 Vaccination

The vaccines available for mpox are the same as those for smallpox. They derive from a mix of two animal viruses, cowpox and horsepox, that evolved through human passage into a unique orthopox virus known as vaccinia. This iatrogenically created vaccinia virus has epitopes in common with smallpox, mpox, and other orthopox viruses, explaining why its use facilitated smallpox eradication.

ACAM2000, a second-generation vaccine developed using modern cell-culture technology, is a clone of vaccinia capable of replicating in mammalian cells. It is administered by pricking the skin using a bifurcated stainless-steel needle dipped into the vaccine [72]. ACAM2000 was developed in the early 2000s and licensed by the US FDA in 2007 [73]. Because ACAM2000 can replicate in our cells, the vaccine virus can spread from the vaccination site to other body parts. Immunocompromised individuals or those with certain skin conditions can develop vaccine-induced vaccinia.

German researchers created the Modified Vaccinia Ankara (MVA) in 1971 from a vaccinia strain kept at the vaccine institute in Ankara, Turkey, and passaged 571 times in chick fibroblasts [74]. This vaccine is a third-generation vaccine and not capable of replicating in mammalian cells. Health authorities selected it during the 2022 mpox PHEIC. MVA was considered safer for an outbreak primarily affecting MSM with a high prevalence of HIV. Bavarian Nordic acquired the rights to MVA technology sometime before 2015; it is marketed as MVA-BN and sold as JYNNEOS, IMVANEX, and IMVAMUNE. Unlike ACAM2000, the MVA-BN vaccines require 2 doses. While studies estimate that 1 dose was 58–64 % [75] effective against disease, the current recommendation is to receive two doses at least 28 days apart, which has been reported to be 89 % effective [76].

The Japanese third-generation vaccine, LC16m8, is a cell-culture-adapted Lister strain of vaccinia that is missing the B5R protein. A single dose is administered using the scarification technique with a bifurcated needle [77]. It generates high neutralizing antibody titers [78].

ACAM2000, MVA-BN, and LC16m8 are recommended by the US CDC, African CDC, and WHO for ring vaccination, with safety considerations as appropriate, to control the 2024 mpox PHEIC. These vaccines have received Emergency Use Authorization (EUA) or Conditional Marketing Authorization (CMA) from Stringent Regulatory Authorities (SRAs) such as the African Vaccine Regulatory Forum (AVAREF), US FDA [79], and WHO.

Additional data on vaccination strategies are highlighted in Table 1D.

**Table 1D:** MPXV treatment and vaccines.

Characteristics management	Clade Ia	Clade Ib	Clade IIa	Clade IIb
Antiviral Treatment to complement supportive care	• Tecovirimat (TPOXX), cidofovir, brincidofovir, and trifluridine (for eye lesions) are used • TPOXX resistance is noted in severely immunocompromised patients • VIGIV can be considered for cases	• Similar treatment options to Ia • Emphasis on managing sexual transmission	• Treated with similar antiviral options as Ia • Emphasis on managing zoonotic transmission	• Similar treatment options • Emphasis on managing sexual transmission • TPOXX resistance developing in immunocompromised patients
Vaccination	• MVA-BN (JYNNEOS, IMVANEX, IMVAMUNE), LC16m8 (Japan), and ACAM2000 • Ring vaccination recommended	• Similar vaccination strategies to Ia	• Similar vaccination strategies to Ia	• MVA-BN is widely used • LC16m8 is used in Japan • ACAM2000 is available under expanded access protocols in some countries • Regulatory frameworks facilitate vaccine approval in Africa (e.g., AVAREF)
Vaccine Efficacy	• Limited data available • Animal models used clade I strains for efficacy studies	• Limited data available	• Limited data available	• Vaccine effectiveness studies ongoing • Vaccinia-binding antibody titers correlate with effectiveness

## 7 The updated Identify-Isolate-Inform (3I) tool for mpox clades Ia, Ib, IIa, and IIb

**Fig. 3:**
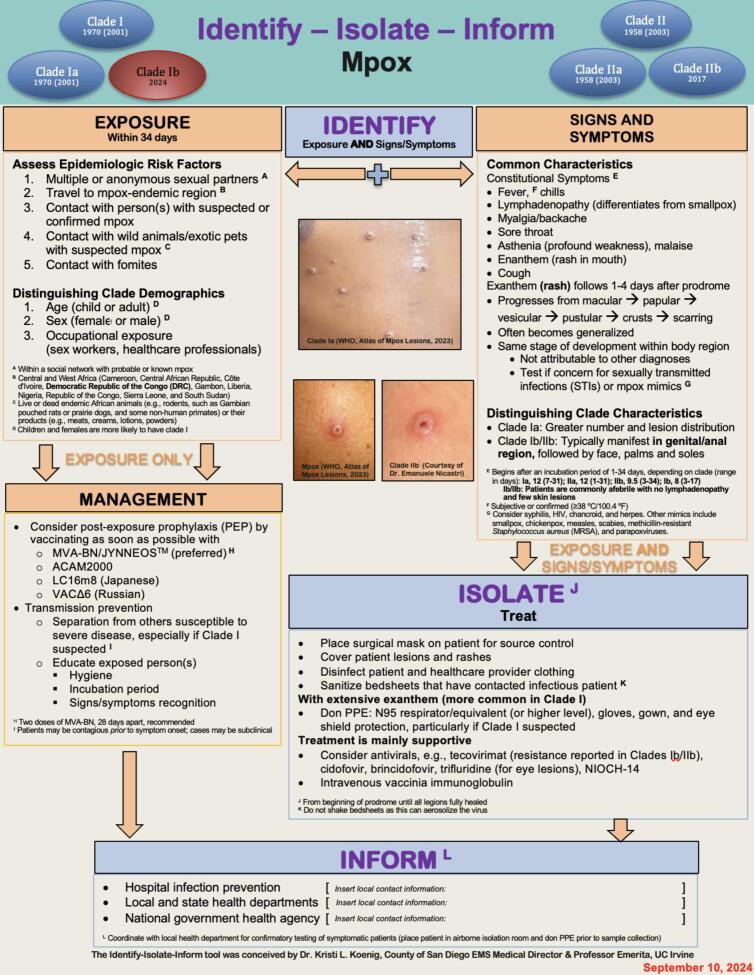
Mpox Identify-Isolate-Inform (3I) Tool.

The management of mpox (previously known as monkeypox) by frontline clinicians is becoming increasingly complex. Particularly noteworthy is the recognition of 1b in 2024 manifesting more commonly in children and females and thought to have a higher mortality rate than Clade IIb, which predominantly affects men who have sex with men. The rapidly expanding global distribution of mpox clades and the disparities in medical countermeasures precipitated a second WHO PHEIC in August 2024. This declaration and the demographic, clinical, and epidemiological distinctions between clades necessitated an updated mpox 3I tool (Fig. 3). The new tool employs the primary approach of *Identify-Isolate-Inform (3I)* [80]. Significantly, it also updates key features and presents a concise visual illustration of the clade classifications.

Clinicians should first *identify* epidemiologic risk factors prior to other vital signs. Application of the “vital sign zero” concept determines the need for PPE [81]. After determination of a potential exposure, the clinician then assesses for signs and symptoms. For asymptomatic patients without physical findings, clinicians should use the “exposure only” algorithm for patient management. For patients with signs or symptoms, the next step is to *isolate*. In either case, clinicians must *inform* hospitals about infection prevention and relevant public health authorities.

Changes from the original Identify-Isolate-Inform 3I Tool [1,82] include:

▪ Name updated from “Monkeypox” to “mpox”
▪ Addition of the clade types and years of presentation
▪ Features that distinguish between clades
▪ Changes to the incubation periods
▪ Revisions to PPE guidelines
▪ Information on antiviral resistance
▪ More recent clinical photos

The 3I Tool has been adapted and applied to numerous existing and emerging infectious diseases, notably COVID-19 [83]. This mpox tool update is important because One Health influences have led to key changes in transmission, risk factors, presentation, and morbidity and mortality. The resulting new knowledge requires translation for clinicians and public health professionals treating individual patients and working to control the global public health emergency.

## 8 Climate change and anthropogenic influences expand mpox outbreak

The warming planet is forcing life on the earth to adapt (or die) in many ways. Heat impacts the physiology and behavior of pathogens, vectors, plants, animals, and humans. It alters the function of mammalian immune systems and, thus, susceptibility to disease. Heat stress alters the hypothalamic-pituitary-adrenal axis, increasing immunosuppressive cortisol levels in humans and animals. [84,85] Elevated ambient heat reduces the conception rate of cows and decreases their energy balance; this negative energy reduces immune function [86].

Loss of vitamins and minerals and a lack of appropriate amino acid intake also negatively impact immune system function. Climatic changes are altering the quality and quantities of foods due in part to floods, droughts, and soil erosion. These impacts on plants, livestock, and other animals are reducing the nutritional value of food [87].

Air, land, and sea transportation networks are facilitating spread of pathogens, including amplifying outbreaks that previously would likely have been extinguished locally when they spilled over in an isolated area. Land use issues, including ground transportation networks (e.g., highways, roads, bridges), are driving humans, animals, and vectors closer together, facilitating more spillover events. The increasing urbanization of the land alters the behavior of wild animals. Urban squirrels have lower vigilance and flight initiation compared to rural squirrels [88], increasing human contact with them.

Each spillover event is an opportunity for a pathogen to adapt to a new host. Pollution, global warming-induced disasters (e.g., fires, cyclones), microplastics, and other waste increase the incidence of non-communicable diseases. These underlying diseases, e.g., cancers and reactive airway diseases, worsen infectious disease risk. The extinction and reduction of many animals and plants force the existing creatures to alter their behavior, often in ways that lead to increased spillover events. These combined forces are driving the zoonotic spread of mpox, particularly clades Ia and IIa. Those clades have adapted into clade b forms that are more easily transmitted from person to person. The development of these more transmissible Ib and IIb that spread with sex and other forms of close human contact reveal that human behavior, travel, and trade are the most direct reasons for the spread of mpox in recent years. Coupled with those issues, vast disparities in access to diagnostic testing, treatments, and vaccines [89] predictably necessitated the 2024 mpox PHEIC.

## 9 Discussion and conclusions

The global expansion of MPXV with the emergence and spread of new clades represents a significant shift in the epidemiology of this amphixenosis. Clade Ia, endemic to Central Africa, and clade IIa, associated with zoonotic transmission, have been joined by the more transmissible Ib and IIb. These newer clades with increased human-to-human transmission largely through sexual contact have expanded MPXV's geographical reach beyond traditional endemic regions.

A complex interplay of factors is driving the rapid change. Anthropogenic changes, including deforestation, urbanization, and climate change, have altered ecosystems and increased human-wildlife interactions, facilitating more frequent spillover events. Climate change has altered the distribution of reservoir hosts, while increased human encroachment into wildlife habitats has created more opportunities for zoonotic transmission. Additionally, regional and global transportation networks and international travel and trade have facilitated the rapid spread of the virus across national borders and continents, challenging traditional containment strategies. At the same time, genetic adaptations, particularly those driven by APOBEC3 enzymes, have enhanced the virus's ability to replicate and transmit in human hosts.

The One Health nature of mpox underscores the interconnectedness of human, animal, and environmental health and shows the importance of equity in reducing the transmission of new diseases. The virus's ability to infect various mammalian species, coupled with its potential for sustained human-to-human transmission, highlights the need for a comprehensive, multidisciplinary approach to disease control and prevention. Collaboration between human and veterinary health sectors, along with environmental scientists and policymakers are crucial for effective surveillance, research, and intervention strategies. There is a need for improved medical countermeasures, education, and technology transfer to underserved areas where spillover of novel pathogens is most likely.

The development of the updated Identify-Isolate-Inform (3I) Tool is crucial in this evolving mpox landscape. This tool provides frontline healthcare workers with a systematic approach to identify potential mpox cases, distinguish between clades, and implement appropriate management strategies. The 3I tool's emphasis on early detection, isolation, and reporting is essential for containing outbreaks and preventing further spread.

This study has several limitations. The rapidly evolving nature of the mpox outbreak means that epidemiological data and genetic information are continually updating, potentially affecting the accuracy of some conclusions. Additionally, the limited availability of clade-specific diagnostic tools and the variability in reporting and surveillance across different regions have undoubtedly led to an underestimation of affected individuals and possibly to some misclassification of cases.

Future research should focus on developing more sensitive and specific diagnostic tools capable of rapidly distinguishing between mpox clades, particularly clade-specific PCR tests. Studies on the effectiveness of current vaccines and antiviral therapies against emerging clades, particularly Ib, are urgently needed. Moreover, there needs to be more equitable distribution of medical countermeasures and global collaboration to foster technology transfer to low-resource areas. Furthermore, investigations into the ecological drivers of mpox transmission and the potential for new reservoir species are essential for predicting and preventing future outbreaks.

Inescapably, mpox is a complex One Health challenge that requires a coordinated, multisectoral response. The updated Mpox 3I Tool is a valuable resource for frontline healthcare workers, but its effectiveness depends on continued surveillance, research, and adaptation to the evolving epidemiological landscape. As we confront the challenges posed by mpox and other emerging zoonotic diseases, a holistic approach that addresses the interconnections between human activities, animal health, environmental changes, and health disparities will be crucial for global health security.

## Disclaimer

Due to the rapidly evolving nature of this outbreak, and in the interests of rapid dissemination of reliable, actionable information, this paper went through expedited peer review. Additionally, information should be considered current only at publication and may evolve as the science develops.

## Funding disclosure

This project did not receive funding.

## Human participant protection

This project did not involve human or animal subjects. Therefore, it did not require institutional review board approval.

## CRediT authorship contribution statement

**Aileen M. Marty:** Writing – review & editing, Writing – original draft, Visualization, Supervision, Formal analysis, Conceptualization. **Christian K. Beÿ:** Writing – review & editing, Writing – original draft, Investigation, Data curation. **Kristi L. Koenig:** Writing – review & editing, Writing – original draft, Visualization, Project administration, Conceptualization.

## Declaration of competing interest

The authors report no conflicts of interest. The views expressed in this paper are those of the authors and do not necessarily represent the views of the institutions with which they are affiliated.

## Data Availability

No data was used for the research described in the article.
